# “The sky is the limit”: adhering to antiretroviral therapy and HIV self-management from the perspectives of adolescents living with HIV and their adult caregivers

**DOI:** 10.7448/IAS.18.1.19358

**Published:** 2015-01-13

**Authors:** Julie A Denison, Harry Banda, Alexis C Dennis, Catherine Packer, Namakau Nyambe, Randy M Stalter, Jonathan K Mwansa, Patrick Katayamoyo, Donna R McCarraher

**Affiliations:** 1 Social and Behavioral Health Sciences, FHI 360, Durham, NC, USA; 2 Department of International Health, Johns Hopkins Bloomberg School of Public Health, Baltimore, MD, USA; 3 FHI 360, Ndola, Zambia; 4 FHI 360, Lusaka, Zambia; 5 Arthur Davison Children's Hospital, Ndola, Zambia

**Keywords:** HIV, adherence, sub-Saharan Africa, adolescents, qualitative, Zambia, antiretroviral therapy, caregivers, youth

## Abstract

**Introduction:**

Worldwide, HIV-related mortality among adolescents living with HIV (ALHIV) increased by 50% from 2005 to 2012 and is attributed in part to a lack of support for adolescent retention to care and adherence to antiretroviral therapy (ART). This vulnerability reinforces the need to better understand incomplete ART adherence among ALHIV, particularly in sub-Saharan Africa, where the majority of the world's 2.1 million ALHIV reside.

**Methods:**

From December 2011 to February 2012, we conducted in-depth interviews with 32 ALHIV (aged 15 to 18) and 23 of their adult caregivers in the Copperbelt Province of Zambia. Interviews were transcribed and translated. An iterative qualitative process was used to code and analyze the data and main themes were summarized regarding the barriers to and facilitators of ART adherence.

**Results:**

More than a quarter of ALHIV reported missing a day or more of ART (ranging from one day to six months). Barriers to ART adherence included fear of disclosure and anticipated stigma. Few youth were willing to take their drugs outside of the home, which led to missed doses of ART. Similarly, families tended to manage HIV within the home only. As a result, although caregivers and families were often the greatest source of emotional and instrumental support, they coped with HIV in isolation of other potential support from their communities, schools or churches. Factors that supported ART adherence included attending clinic-sponsored youth groups, wanting to maintain one's health and using phone and clock alarms. Involvement of adult caregivers in HIV management varied greatly and was often based on the age and health status of the youth. Some caregivers struggled with letting the adolescents assume responsibility for their medication, and ALHIV had few self-management skills and tools to help them regularly take ART.

**Conclusions:**

These data highlight the importance of families and home environments in supporting adherence to ART among ALHIV. Skill-building and family-based interventions to prepare ALHIV and their adult caregivers for HIV self-management and HIV status disclosure by youth are of paramount importance. Future research and programmes also need to address the fears adolescents and families have regarding HIV-related stigma that shape young peoples’ adherence behaviours.

## Introduction

Globally, an estimated 2.1 million adolescents are living with HIV (ALHIV), with the majority located in sub-Saharan Africa (SSA) [1]. These adolescents are both perinatally and behaviourally infected youth who are maturing into adulthood. Despite an international call to support HIV self-management among adolescents [2–4], including adherence to antiretroviral therapy (ART), ALHIV and their caregivers in SSA often lack access to adolescent-specific services [2] and the skills needed to manage a highly stigmatized chronic illness [5–13]. Moreover, HIV programmes in SSA are often clinic-based and focus on individual medical outcomes, with few available resources to address the social, familial and stigmatized context in which ALHIV live. This gap in HIV services is illustrated by the 50% increase in HIV-related deaths among adolescents worldwide from 2005 to 2012, while the overall global number of deaths across age groups decreased by 30% [2]. The World Health Organization (WHO) attributes this growth in mortality in part to “a lack of support for adolescents to remain in care and to adhere to antiretroviral therapy” [2, p. viii].

Results from the limited number of adherence studies conducted among children and adolescents in SSA have often found sub-optimal adherence to ART and poor virologic outcomes [14–18] and tend to focus on youth below the age of 15 [17,19–21]. In Zambia, research and donor reporting requirements have historically grouped ART patients aged ≥15 with adults and <15 with children, making it challenging to assess the numbers and clinical status of ALHIV [6]. Similarly, a meta-analysis found only eight eligible studies from SSA that generated a pooled adherence estimate of 83.8% (95% CI 78.9–88.7) among SSA youth, compared to 62% (95% 57.1–67.6) from youth across all regions [22]. While this high level of adherence is encouraging, four of the SSA studies included young adults aged up to 24 [23–25] and 28 [26] and two that disaggregated their findings by age concluded that adolescents had lower viral load suppression than adults [25,26]. Only one study to date, conducted among 15 Ugandan adolescents, has assessed adherence using electronic monitoring with less than 25% achieving >95% adherence over a year [15]. Overall, these data underscore the importance of assessing and understanding adherence to ART among ALHIV. This need is particularly pressing in countries such as Zambia where an estimated 12.7% of adults (15 to 49 years) and 160,000 children (<14 years) are living with HIV [1]. These children have the opportunity to mature into adolescence and adulthood and to become responsible not only for managing their ART but also the prevention of secondary HIV transmission.

This study used qualitative methods to explore ART adherence from the perspectives and experiences of older ALHIV (aged 15–18) and their adult caregivers in Zambia. Despite literature on the importance of families in managing chronic illnesses [27–34] and the stress experienced by families who care for orphans and vulnerable children [35], few studies have included caregivers in their examination of adherence among ALHIV. It is critical to understand adolescents’ and adult caregivers’ experiences with these issues because older ALHIV are at an age when they begin to assume greater responsibility for their HIV care, treatment and prevention behaviours.

## Methods

The study was conducted in two ART clinics in Ndola, Zambia, from December 2011 to February 2012. One clinic was in a children's hospital and the second in a central hospital. Both clinics offered adolescent-specific clinic days and monthly youth support groups. We purposefully selected adolescent clients aged between 15 and 18 attending these two ART clinics. Initially, clinic staff selected adolescents; this raised concerns among the study team that only patients emotionally close to the providers, or those who may be highly adherent, would be invited to participate. To capture a variety of experiences, the team randomly selected youth attending the clinic on a given day. All consenting adolescent participants were invited to complete two in-depth interviews (IDI) that lasted approximately one hour each; the first interview focused on participants’ experiences with HIV care and treatment, including ART adherence, and the second interview focused on the participants’ sexual and reproductive health needs. With the adolescents’ permission, their adult caregiver was invited to participate in an IDI. Caregivers were defined as adults over the age of 19 who knew the adolescents’ HIV status, helped care for the youth and were the adult contact for the clinic. All caregivers underwent a written informed consent process. A semi-structured field guide was used with open-ended questions and suggested probes (e.g. “tell me about the last time you missed taking your ARV drugs.”). Young Zambian adults with research or youth programme experience were trained to conduct the interviews and pre-tested the field guides. Interviews were conducted in Bemba, Nyanja or English and were audio recorded.

### Data analysis

All interviews were transcribed verbatim, translated when necessary into English, reviewed for accuracy and entered into NVivo v.8 (QSR International). An iterative process was used to analyze the data [36] with an initial codebook developed based on the interview guides and preliminary data and revised as the analysis team identified new themes. Fifteen percent of the youth and caregiver transcripts were coded independently by at least two coders (HB, JAD and CP) and discrepancies were discussed and resolved to ensure inter-coder reliability. The codebook was updated as a result of these discussions and as the analysis team identified new themes. Coding reports were generated from NVivo. We then developed matrices for both male and female participants and wrote memos summarizing the main themes of each coding report. The analysis presented here focuses on the themes related to ART adherence (Table 1).

**Table 1:** Emerging themes regarding the barriers and facilitators to antiretroviral therapy (ART) adherence and transitioning to self-management among 15- to 19-year-old adolescents living with HIV (ALHIV) and their caregivers

**Barriers** ALHIV and caregivers felt that knowledge of an adolescent's HIV status should remain in the home and feared unintentional disclosure if an ALHIV is seen consuming ART outside of the house. Not being home at dosing time was the most common reason for not taking one's ART. Religious healing led to incomplete ART adherence **Facilitators** The families of ALHIV reminded youth to take their medication. This support ranged from occasional reminders to direct observation of swallowing the pills. ALHIV youth groups provided friendship, encouragement and motivation to continue taking ART. ALHIV adhere to ART so that they can be physically healthy as the majority initiated treatment after prolonged illnesses and attribute their return to health to ART. **Transition to HIV self-management** Transitioning to self-management was related to both the health and the age of the ALHIV with some caregivers struggling with letting youth assume greater responsibilities for their medication. Youth used watches, clocks and phone alarms to remind themselves of dosing time.

### Ethical considerations

FHI 360's Protection of Human Subjects Committee, the Eres Converge Institutional Review Board in Zambia and the Zambia Ministry of Health reviewed and approved this study. All interviewers underwent a seven-day training session covering research ethics, study objectives and procedures. Confidentiality was maintained by conducting the interviews at the clinic in private rooms. The ethical review boards granted permission to waive parental consent for adolescent participation.

## Results

### Participant characteristics

Sixteen males and 16 females on ART (aged 15–18) were interviewed and all but one completed both IDIs for a total of 32 adolescent participants and 63 transcripts. Self-reported modes of HIV acquisition included perinatal (*n*=26), behavioural (*n*=4) and unknown (*n*=2). Adolescents self-reported taking their first HIV test at age 13 on average (ranging from 11 to 18 years) and became aware of their HIV-positive status at age 14 on average (ranging from 12 to 18 years). Twenty-eight of the 32 participants were currently attending school.

Twenty-three of the adolescents’ adult caregivers were also interviewed with an average age of 43 (range 23 to 70 years). Nineteen were female (aunts, mothers, sisters, grandmothers and stepmothers) and four were male (father, cousin and uncles). Nine adults who declined to participate cited work, distance to clinic and being too busy, while several caregivers also expressed concern about being seen at an HIV clinic and potentially labelled as living with HIV themselves.

### Barriers to ART adherence

Twenty of the 32 participants discussed missing doses of ART. More than a quarter of all interviewed youth experienced ART interruptions ranging from one day to six months. Below, we present the main reasons adolescents and their caregivers gave for missing ART.

#### Fear of unintended disclosure

Fear of unintentionally disclosing the youth's HIV status emerged as the most salient barrier to ART adherence. The majority of adolescents and adults felt that knowledge of an adolescent's HIV status should be kept within the home and within the family. These views corresponded with adolescents’ reports of keeping and taking their drugs in their homes to avoid being seen with their HIV medication and possibly being labelled as HIV positive. As described by one 17-year-old female participant, disclosing to friends meant, “I will be segregated and they will only want to play with those who are not HIV positive.” An aunt reported, “There is nobody who knows [her nephew's HIV status] … it is just known within the family.”

This need to keep their status a secret was directly linked to incomplete adherence. Social and school events frequently kept youth from their homes at dosing times, resulting in missed ART. For example, youth who missed doses when playing were concerned that disrupting a game to go home to take their medication would generate unwanted questions from their peers who were unaware of their serostatus. Thus, adolescents often felt they had to choose between adhering to ART and keeping their HIV status a secret, and some navigated this situation better than others. One 16-year-old male participant described his reaction when asked to join a football match at dosing time: “you should tell your friends that, guys, I will be coming [to play football] first I am on medicine … [but] my friends do not know that I have the virus [so I said] I felt some malaria.” The adults echoed this sentiment, as exemplified by a stepmother who said, “You can never give her the drugs to take at school in the presence of her friends.”

While youth often delayed taking their medication for several hours when playing with friends, a more common reason for missing one's dose for a day or more was travelling away from home without ART. Again, the underlying reason for not travelling with enough medication was fear of unintended disclosure: “I sleep over and then I forget to get extra [ART] because I don't want the people that I am staying with to know that I am taking them” (female participant, 18 years).

Being away from home and fear of disclosure were also the most frequent reasons adult caregivers cited for their adolescent's missed ART doses. One mother said: “the time I sent him for a holiday for just three days, he never took his drug—reason being he was feeling uncomfortable around his friends.” In reaction to this situation the youth's mother said, “I had to step my foot down and say no more holidays as long as you are still irresponsible.” Other caregivers discussed similar concerns about their youth's ability to take drugs outside of the home, “I don't allow anyone to take this child … even the family members from their biological mother … they can just come and visit and go” (stepmother).

#### Spiritual beliefs and healings

Spiritual beliefs also played a role in incomplete adherence. One 18-year-old female participant stopped taking her ART for six months after being declared healed by adults at a healing prayer school in South Africa. The adult caregivers also shared experiences of ALHIV becoming non-adherent after attending a: “… church where … the pastor started praying that all those who take ARVs have been healed …. So that's how come he [my nephew] stopped taking the medicine” (aunt). A grandmother discussed how she started her granddaughter on ART again after the clinic staff explained why a healing may not have worked: “… we were told not to stop children from taking the drugs because even the faith of being healed is not yet in them. You would think she is healed but her personal faith is not matching with what is required.”

#### Other reasons for incomplete adherence

Other reasons for incomplete adherence included not knowing one's HIV status, side effects and not wanting to take medication daily. An 18-year-old female participant said, “I didn't know what this medicine is for … sometimes I used to drink, sometimes I used [to] throw [it away] … since I didn't know.” Similarly an aunt discussed how her niece stopped taking her medication and when confronted, her niece said, “I don't even know why I am given this medicine.” Two other female participants discussed how ART would make them “feel sick” and make them oversleep, leading to missed doses: “When I want to drink [ART] I would [say] not today, I won't take as I have to wake up to study … if I don't … I'll fail … so I used to miss that [dose].” Adult caregivers also discussed how youth often simply forgot to take their medication; some adults attributed the forgetfulness to the young person's age, while others cited memory loss as a side effect of ART. An uncle said, “We are talking about a very brilliant kid … but … after two years of taking these drugs, these drugs have tampered with her memory.” Another 18-year-old female participant said that taking one's medication is “tiresome” but necessary, and a male participant asked the interviewer “are we going to take this medication forever?”

### Facilitators of ART adherence

Youth also shared the kinds of support and tools, including instrumental and emotional support, that helped them routinely adhere to ART.

#### Family support

The majority of youth discussed how their families reminded them to take their medication. These reminders were sometimes inconsistent and depended upon whether the family member lived with or visited the youth frequently. While many youth appeared to appreciate these reminders as a sign of caring, one 18-year-old female participant said, “Grandma reminds me but … she doesn't know whether …. I really take them or I don't.” Other participants shared how their family provided more hands-on support by waking them up to take the drugs, giving them ART to swallow or checking the number of pills remaining in a prescription. An uncle shared how the family “developed a medication chart … at home where my wife, my young sisters, everyone knew that at given times we are to give medicine to this child.”

In addition to verbal reminders, many participants discussed how their families provided them with emotional support and reasons to take the medication. For example, one 18-year-old female participant described how her grandmother and mother counselled her: “They told me that it doesn't mean that when you are HIV-positive then that is the end of the world no, you can still continue living … you can still continue doing what you used to do.” A 16-year-old male participant shared how his parents encouraged him to: “drink this medicine for we really want you to be someone in life.” An uncle stated “if you are consistent in taking your medicine you will be able to stabilize and the sky is the limit.”

#### Clinic youth support groups

Overall, adolescents spoke of the clinic-sponsored groups for ALHIV as a valuable opportunity to make friends, encourage their peers and help each other remember to take their medications and hear about the experiences of other ALHIV. One 16-year-old male participant said, “… when I am at home maybe I feel like I am the only person who takes the medicine … but when I come here I see a lot of people who take this medicine … and who really care about the same medicine I wanted to stop [taking].” The youth continued to describe how seeing his peers helped change his attitude towards ART, until he decided “I will go and take my medicine.”

#### Wanting to live longer, healthier lives

When asked specifically why they take their ART, adolescents overwhelmingly said because they do not want to be sick or die. The majority of youth initiated ART after prolonged illness and experienced vast improvements in their health, which they attributed directly to ART. One 18-year-old male participant said, “How I am now and how I used to be is different.” Another female participant said she took the drugs “because I want to live longer …. I want to be healthy and not get sick because I already know when I don't take them … what can happen … rash … diarrhea, blood … a lot of things.” A 17-year-old female participant exemplified a shift in thinking over time: “At first when I started drinking the medicine … aah … I was just drinking … just to get well. But now I drink so that I can live longer.”

### Transition to HIV self-management

Only a few youth explained how they started assuming responsibility for taking their ART. One female participant said, “At first [my grandmother] is the one who used to keep the medicine. So each time she realizes that it is time to take medicine, she would tell me. But when I started feeling much better, they gave me the medicine to keep … they have stopped reminding me” (age 18). A 16-year-old male participant also discussed how his family “used to remind me when I was young but now they have told me that since you have grown we don't need to be reminding you, you have to be taking medicine on your own.” As these examples illustrate, transitioning to self-management appears related to both the health and the age of the young person. However, some caregivers struggled in letting youth assume responsibility for their medication. One stepmother who kept her step-daughter's medication in her bedroom said, “I think at the stage she has reached, she can manage to take the drugs even if they were in her bedroom … but I don't just like to take chances she might not take them … so that is what has stopped me from taking the drugs to her bedroom despite her being quite old now, I will still continue giving her [the medication].”

While transitioning to self-management was not frequently discussed, several youth talked about how they remembered to take their medication. One 16-year-old male participant reported taking his drugs at the same time in the morning and evening “like a habit.” Similarly, about a third of youth reported using a phone, watch alarm or clock as tools for remembering to take their medication. One 18-year-old female participant said, “Especially if I am really busy with school or anything, I set an alarm so that I can remember coz I can easily forget to take my drugs.” Two 17-year-old male participants also shared how they used alarms at school: “… when I am wearing my watch say in class the alarm would go off … [I would] go out [to] take the drugs.” An aunt discussed how she “put an alarm on his [nephew's] wrist watch … when he knows now he is going … he would pick the drug and put it in a container and put it in his pocket so that when the alarm goes off, wherever he is he will be able to take the drugs.”

## Discussion

To our knowledge, this is among the first qualitative studies to specifically focus on the ART adherence experiences of older Zambian adolescents and their caregivers and emphasizes the important role that families have on the medication taking behaviours of youth. Data were analyzed separately for males and females; however, we found that the main themes detailed above did not differ by sex among this group of predominately perinatally infected ALHIV. Fear of stigma and unintended disclosure were the salient themes shaping both the barriers to and facilitators of adherence across age, sex and living situations. Overall, fear of disclosure discouraged most adolescents from consuming pills outside of their homes, which resulted in missed ART. Similarly, families tended to manage HIV within the home only. As a result, caregivers and families were often the greatest source of emotional and instrumental support but coped with HIV in isolation of other potential support from their communities, schools or churches. In the few cases where adolescents or caregivers did talk about church or religious leaders, it was in the context of the adolescent no longer taking ART following a religious healing. Churches and faith-based organizations represent important religious and social organizations within Zambia and remain an untapped resource in supporting ALHIV and their families.

The study findings also highlight the importance of maintaining one's health as a motivator for ART adherence among ALHIV. Most participants were severely sick when they started ART. With the adoption of the new WHO recommendations to treat adolescents when their CD4 is ≤500 cells/mm^3^ [37], more adolescents may initiate ART when they are experiencing fewer symptoms. It will be important for programmes to examine if adherence and motivation to adhere differ for ALHIV when they start ART when healthier.

Overall, these findings corroborate other research that emphasized the importance of families, psychosocial environments and caregiver characteristics on ART adherence and well-being of younger children living with HIV in SSA [6,17,38,39] and among perinatally infected youth in the United States [40–42]. While the need for caregiver involvement is undisputed when discussing medication adherence among children and young adolescents (<14), the role of families and caregivers has been explored less in relationship to older ALHIV. What our research shows, however, is that efforts to improve HIV self-management need to involve not only the adolescent but also their caregivers/families. Such efforts also need to address the fears families have regarding HIV disclosure among ALHIV and anticipated stigma.

### Implications for future research

Future research should quantitatively assess how family involvement and characteristics influence the HIV self-management behaviours of ALHIV. Our study also supports the development and testing of family-centred approaches for working with ALHIV. Potential intervention strategies include holding group sessions not only with older ALHIV but also separately with their adult caregivers. Peer groups have improved adolescent self-management of various chronic illnesses, including diabetes, asthma [30] and HIV [2,21], and have shown promise in reducing HIV-related stigma among adults in SSA [43]. Evaluations of such interventions should examine if working with the adult caregivers have an added impact on the HIV self-management behaviours of ALHIV above and beyond what may be achieved working with the adolescent alone. Such interventions may also provide a safe space for discussing fears around HIV status disclosure and allow families to develop strategies and skills to support the on-going decisions adolescents will need to make regarding disclosure as they transition into adulthood.

### Limitations

This study took place in an urban setting, and the majority of participants self-reported being perinatally infected, were enrolled in school and attended monthly ART clinic youth groups. As such, we cannot extrapolate the findings to other ALHIV in Zambia, including ALHIV who are behaviourally infected or live in rural areas. Findings from caregivers also may not be transferable to caregivers who chose not to participate. Despite these limitations, these data provide unique insights into the contextual challenges ALHIV encounter as they transition into adulthood and manage their own treatment of HIV.

## Conclusions

These data highlight the importance of families and home environments in supporting and hindering ALHIV adherence to HIV. Skill-building and family-based interventions to prepare ALHIV and their adult caregivers for HIV self-management and HIV status disclosure by youth are of paramount importance. These programmes also need to address the concerns and fears of HIV-related stigma that adolescents and their families experience and that shape young peoples’ adherence behaviours.
